# Internet use in disease management for home care patients: A call for papers

**DOI:** 10.2196/jmir.4.2.e6

**Published:** 2002-11-25

**Authors:** George Demeris, Gunther Eysenbach

**Affiliations:** ^1^University of Missouri-ColumbiaHealth Management and InformaticsMissouriUSA; ^2^University of TorontoDepartment of Health Policy, Management and EvaluationTorontoCanada

## Disease Management and the Internet

Disease Management refers to "a set of coordinated healthcare interventions and communications for populations with conditions in which patient self-care efforts are significant." [1] Disease management supports the care plan and enhances the provider-patient relationship. It emphasizes prevention of deterioration and/or complications using evidence-based practice guidelines. It aims to improve the patient's overall health by continuously assessing clinical and economic outcomes. The goals of disease management are to manage medical conditions over time, improve outcomes, lower costs, and support patient-provider interaction, patient education and monitoring.

Patients with chronic illnesses account for a great portion of healthcare costs. An efficient disease management system should dramatically reduce medical and administrative costs, while enriching the physician-patient communication and improving health outcomes.

Earlier efforts in utilizing information technology demonstrated the barriers of attempting to integrate systems without a common protocol and developing systems with a long implementation cycle and at increased overall costs. However, the diffusion of the Internet has the potential to empower patients and address these barriers by providing the means for technically flexible applications with shorter implementation cycles.

Internet technologies are being utilized for disease management in many clinical areas in the last few years. In Textbox 1 some examples of web-based disease managementapplications are provided, grouped by clinical area. Internet technologies allow to connect patients with providers, link home-care with hospital and ambulatory care, facilitate information exchange, communication, and collaboration between and among patients, caregivers, and health care providers. Patient self-management education is a central component of disease management, and the Internet supports this by enabling the transmission of tailored health information or automated reminders to patients or their caregivers. Web-based electronic health records are another avenue of enhancing communication among stakeholders to coordinate care, and patient accessible records empower patients to improve self-care in the age of consumer health informatics [2]. The convergence of the Internet with everyday household items such as TV sets, refrigerators, Personal Digital Assistants (PDAs) and mobile phones [3] opens up new channels of communicating with patients through information technology and empowering them to manage their disease.

## Challenges for web-based disease management systems

Factors that will be critical for the diffusion of Internet based disease management systems include design; privacy and confidentiality; patient and provider acceptance; costs and reimbursement structures; and access to and ownership of data.

### Usability

A great number of home care patients who require disease management are elderly and/or have functional limitations. A functional limitation describes a "reduced sensory, cognitive or motor capability associated with human aging, temporary injury, or permanent disability that prevents a person from communicating, working, playing or simply functioning in an environment where other people in the population can function." [11]. Although the Internet seems to have the potential to revolutionize the process of health care delivery and empower patients to become more active in the care process, the fastest growing segment of the US population — i.e., people over the age of 50 years — are at a disadvantage because designers of both software and hardware technology fail to consider them as a potential user group. Usability and accessibility issues are important quality criteria for web-based interventions, but are frequently ignored by designers and evaluators [12]. The design of a usable web-based information system for healthy users who are familiar with computer technology is a challenge. When a system needs to address age-related constraints and the functional limitations of inexperienced users, it becomes even more difficult. Designers of a system for home care patients should aim to increase its functional accessibility [13] and employ rigorous usabiliy testing methods.

**Examples of web-based disease management applications**

Asthma management

Disease management for asthma patients has the potential of early detection of critical situations and timely intervention.

One example of Internet utilization for asthma management is the home asthma telemonitoring (HAT) [4] system which provides patients with continuous individualized help in the daily routine of asthma self-care and notifies health care providers if certain clinical conditions occur.

Diabetes management

Diabetes has in many cases an asymptomatic nature. The time frame between sustained hyperglycemia and observable complications can be extended, thus making a long-term program of secondary prevention an essential part of appropriate diabetes care. The Center for Health Services Research, Henry Ford Health System in Detroit, Michigan, developed a Web-based Diabetes Care Management Support System (DCMSS) to support the provision of routine care to patients with diabetes [5]. The system was evaluated within a nonrandomized, longitudinal study and findings suggest that web-based systems of clinical practice guidelines, patient registries, and performance feedback have the potential to improve the rate of routine testing among patients with diabetes. McKay and colleagues studied the development and feasibility of a Web site for diabetes self-management that emphasized personalized goal setting, feedback, and social support [6]. The Telematic Management of Insulin-Dependent Diabetes Mellitus (T-IDDM) project, funded by the European Union, piloted, implemented and evaluated a distributed computer-based system for the management of insulin-dependent diabetes mellitus. The goal of this system is to utilize Internet technology to support the normal activities of the physicians and patients involved in the care of diabetes by providing them with a set of automated services ranging from data collection and transmission to data analysis and decision support [7]. The system includes a module allowing patients to automatically download their monitoring data from the blood glucose monitoring device, and to send them to the hospital data-base. The system provides physicians with a set of tools for data visualization, data analysis and decision support, and allows them to send messages, including therapeutic advice, to the patients [8].

Post-Transplant care

Regular spirometry monitoring of lung transplant recipients is essential to early detection of acute infection and rejection of the allograft. A prospective study investigated the impact of a user-friendly, Internet-based telemonitoring system providing direct transmission of home spirometry to the hospital. The authors concluded that home monitoring of pulmonary function in lung transplant recipients via the Internet is feasible and provides very reproducible data; yet "it has only a mild sensitivity for the detection of acute allograft dysfunction." [9].

Wound care

The TeleHomeCare Project at the University of Minnesota utilized low-cost commercially available monitoring devices and Internet access to enable congestive heart failure, chronic obstructive pulmonary disease and wound care patients at home to interact with health care providers at the agency. Individualized web pages were designed for each patient including a diary system with questionnaires to be filled out daily. The questionnaire included questions about vital signs (such as weight, blood pressure or temperature), symptoms, and overall well-being and nutrition. Alerts were triggered when a patient's response required immediate medical attention according to predefined rules [10].

### Privacy and Confidentiality

The healthcare sector worldwide is facing a great number of challenges and regulations in regard to the confidentiality, availability and integrity of individual health information. In the United States, the Notice of the Proposed Rule from the Department of Health and Human Services concerning Security and Electronic Signature Standards was introduced in 1998 [14]. The Proposed Rule falls under the umbrella of the Health Insurance Portability and Accountability Act (HIPAA) that was passed in 1996. This Proposed Rule became law in 2000 in the United States and suggests standards for the security of individual health information and electronic signature use for health care providers, systems and agencies. These will use the Security Standards to develop and maintain the security of all electronic health information. Similar frameworks exist in the European Union and Canada [15].

### Patient and provider acceptance

The diffusion of an innovation depends to a great extent to the attitudes of the population to which it is being introduced to. This of course applies to web-based disease management applications as well where users (patients, caregivers, family members, providers) have to accept the use of technology and be willing to receive training and integrate the application into the care delivery process.

### Costs and reimbursement

While there is some evidence demonstrating the cost-effectiveness of traditional disease management (e.g., a retrospective analysis of 7,000 patients found a $50 per member, per month savings in diabetes treatment costs over twelve months and eighteen percent decrease of admissions [16]) there is little evidence as of yet of the cost-effectiveness or even possible long term cost reduction through utilization of Internet in disease management. Cost analysis and/or cost-effectiveness studies will contribute to discussions about possible reimbursement issues of web-based monitoring services and the question of which party will bear the costs of implementing and maintaining such a web-based system.

### Access to and ownership of the data

In many web-based applications in home care, patients enter or record monitoring data and transmit them daily to a web server owned and maintained by a private third party that allows providers to log in and access the data of their patients. The question of patients' rights to access parts or all of their record, the physical storage and access rights and the issue of data ownership become even more essential when monitoring data are stored physically at a separate location controlled by a private company. The implications are not only possible threats to data privacy but extend to ethical and political debates about restructuring the care delivery process and introducing new key players.

## Call for papers

The *Journal of Medical Internet Research* is pleased to announce a theme issue on Internet utilization for disease management in home care. We invite researchers in this field to submit papers that focus on this area such as:

- Studies (preferably randomized controlled trials) that demonstrate the impact of Internet utilization in disease management on
  - health outcomes
  - patient self-management education
  - cost of care
- Papers that describe the development and evaluation of web-based disease management applications
- Studies that address design issues for such applications
- Studies that describe innovative web-based patient monitoring systems and/or devices (an evaluation component is strongly encouraged)
- Studies that propose a sustainable and cost-effective model for web-based disease management
- Manuscripts that address the issues of privacy and confidentiality of patient data (e.g. the impact of final HIPAA privacy rule on disease management via Internet for the US)
- Critical comments and opinion papers
- Systematic reviews synthesizing our current state of knowledge in this field

All papers will undergo a normal peer-review process. Papers received before June 1^st^, 2003 will have the best chances for publication. The theme issue is planned to appear in late 2003. We will be actively looking for a sponsor of this theme issue, which will enable us to waive our usual article processing fee for papers published in this theme issue. The theme issue will be Medline-indexed and be made freely accessible on the web and possibly in a printed version.

George Demiris, PhD

Assistant Professor, Health Management and Informatics, University of Missouri-Columbia

DemirisG@health.missouri.edu

Guest Editor, *J Med Internet Res*, Theme Issue "Disease Management and the Internet"

Gunther Eysenbach, MD

Associate Professor, Department of Health Policy, Management and Evaluation, University of Toronto

Editor, *J Med Internet Res*
